# LncRNA Rik-203 Contributes to Sevoflurane Induced Neurotoxicity?

**DOI:** 10.3389/fmed.2020.00353

**Published:** 2020-07-22

**Authors:** Lei Zhang, Zhenyu Xue, Jia Yan, Hong Jiang

**Affiliations:** Department of Anesthesiology, Shanghai Ninth People's Hospital, Shanghai Jiao Tong University School of Medicine, Shanghai, China

**Keywords:** anesthesia, sevoflurane, Rik-203, miR-466l-3p, BDNF, neural differentiation

## Abstract

**Background:** The anesthetics inhibit neural differentiation, induce neuron loss and cognitive impairment in young animals. However, the underlying mechanisms of anesthesia on neural differentiation are unknown.

**Methods:** Embryonic stem cells (ESCs) and mice received sevoflurane anesthesia. RNA sequencing; gene expression of mRNAs, LncRNAs and miRNAs; over-expression and RNA interference of genes; flow cytometry; real-time quantity PCR and Western blot were used in the studies. RNA pull-down assay and PCR were employed to detect any miRNA that attached to Rik-203. The binding of miRNA with mRNA of BDNF was presented by the luciferase assay.

**Results:** Here we found that LncRNA Riken-203(Rik-203) was highly expressed in mice brain and was upregulated during neural differentiation. Sevoflurane decreased the amount of Rik-203 in mice brain. Knockdown of Rik-203 repressed the neural differentiation derived from mouse embryonic stem cell and downregulated the neural progenitor cells markers Sox1 and Nestin. RNA pull-down showed that miR-466l-3p was highly bound to Rik-203. Inhibition of miR-466l-3p restored the neural differentiation repressed by Rik-203 knockdown. Brain derived neurotrophic factor (BDNF), which was downregulated by sevoflurane, was also directly targeted by miR-466l-3p. Overexpression of BDNF restored the neural differentiation repressed by miR-466l-3p and Rik-203 knockdown.

**Conclusion:** Our study suggested that sevoflurane related LncRNARik-203 facilitates neural differentiation by inhibiting miR-466l-3p's ability to reduce BDNF levels.

## Introduction

The widespread and growing use of anesthesia in children makes its safety a major health issue of interest (1), reviewed in (2). It has become a matter of even greater concern as evidence shows that multiple exposures to anesthesia and surgery may induce cognitive impairment in children (3–8), and that anesthetics may induce neurotoxic damage and cognitive impairment in young animals (1, 9–13). Several clinical studies on anesthesia/surgery-induced cognitive impairment in children have been reported (3–8). However, contradictory reports also exist (8, 14, 15), and single and short time exposure to anesthesia and surgery is not associated with cognitive impairment in children (16, 17). Nevertheless, these findings suggest that children who have undergone anesthesia and surgery may not develop to their full cognitive potentials.

Aberrant neural differentiation has been shown to contribute to cognitive impairment and neurogenesis inhibition in young and offspring rodents (18). Sevoflurane regulates neurogenesis in offspring rats by down-regulating the expression of brain-derived neurotrophic factor (BDNF), which is the critical neural development and disease related gene (19–22) and induces neurotoxicity and cognitive impairment in young mice (23, 24). But, the underlying mechanism by which sevoflurane regulates the expression of BDNF remains largely unknown, which impedes further research into anesthesia neurotoxicity in the developing brain. In the present study, we set out to determine the effects of sevoflurane on neural differentiation and the underlying mechanisms of sevoflurane-regulated expression of BDNF.

Long non-coding RNAs (LncRNAs) are defined as transcripts that are longer than 200 nucleotides. They are reported to be critically involved in the regulation of neural differentiation (25) and are associated with sevoflurane anesthesia (19, 26). LncRNAs, e.g., NBAT-1 and Pnky, may regulate cell differentiation and development [(27–29), reviewed in (30)]. LncRNA Rik-201 plays important role in gliomagenesis (31). In our previous study, LncRNA Rik-203 contributes to anesthesia induced neurotoxicity by regulating neural differentiation (26). Moreover, LncRNA Rik-201 and LncRNA Rik-203 attached to microRNAs (miRNAs) as a sponge to prevent the miRNA from binding to mRNA 3′UTR, thus inhibiting miRNA's ability to bind to target mRNA and prevent the translation to regulate neural differentiation (32).

In this study, we systematically investigated the interaction between LncRNA Rik-203, the anesthetic sevoflurane, miRNA and the brain-derived neurotrophic factor (BDNF). As a result, sevoflurane inhibited neural differentiation by down-regulating the expression of LncRNA Rik-203. Our study suggested an important role of LncRNAs in the underlying mechanism of the anesthesia neurotoxicity.

## Materials and Methods

### Cell Culture

46C mESCs were cultured in Dulbecco's Modified Eagle Medium (DMEM) (Hyclone, USA) supplemented with 15% fetal bovine serum (Gibco, USA), 55 μM β-mercaptoethanol (Thermo, USA), final concentration 1:10,000 leukemia inhibitory factor (Millipore, USA), 1% nonessential amino acids (Thermo, USA), 1% L-glutamine (Thermo, USA) and 1% sodium pyruvate (Thermo, USA) at 37°C, 5% CO_2_ atmosphere. 46c mESCs is a Sox1-GFP reporter ESCs line that recapitulates endogenous Sox1 expression when GFP is expressed. Quantification of the Sox1 positive cells using fluorescence microscopy showed the inhibition of neural differentiation.

### Inducible Rik-203 Knockdown 46c Cell Lines

We constructed two shRNA vectors for targeting different sites by using plko-tet-on vector. The sequence of shRNA is as follows: shRNA-1, 5′-GGTGTTGGGCCAGTTCCTTAT-3′; shRNA-2, 5′-GCTTGAATTCAGGCTGCTTGA-3′.

For knockdown of the Rik-203, mESCs were dissociated with 0.05% trypsin and infected with rtTA lentivirus supplemented with 8 × 10^−3^ μg/mL polybrene (final concentration 1:1,000). After 48 h later, cells were treated by final concentration of 200 μg/ml geneticin G418 (Thermo, USA) to select stable transfected cell line. Selected cells then were infected by plko-tet-on lentivirus for 48 h with 8 × 10^−3^ μg/mL polybrene before selection with 5 μg/mL puromycin (Sigma-Aldrich, USA).

### Neural Differentiation From mESCs

mESCs were dissociated to single cells with 0.05% trypsin (Hyclone, USA) and then neutralized with DMEM (Gibco, USA) with 10% fetal bovine serum (Gibco, USA). mESCs were washed with GMEM (Gibco, USA). Cells were resuspended and cultured into a low adsorption petri dish (Thermo) with neural differentiation medium at the density of 2.5 × 10^4^ cells/ml. The neural differentiation medium contains GMEM with 8% knockout serum replacement (Gibco, USA), 1% L-glutamine, 1% sodium pyruvate, and 55 μM β-mercaptoethanol. The medium was changed everyday until 7 days. The single-cell clone could be identified under microscope.

### Overexpression of miR-466l-3p

The plvx-puro-miR-466l-3p vector (Biogot technology,co,Ltd.,China) lentivirus was infected with the 46c mESCs to establish the miR-466l-3p overexpressing cell line.

### Inhibition of the miR-466l-3p

Lipofectamine 2000 (Thermo) was used to tansfect the miR-466l-3p inhibitor, a chemically modified RNA single chain competing with mature miR-446l-3p, or control inhibitor (Ribobio,China) following the instructions to inhibit the function of miR-466l-3p at day 3 and day 5 during the neural differentiation.

### Overexpression of BDNF

The whole RNA was isolated by the RNAiso plus (TaKaRa, China) and inversed transcription to cDNA by cDNA Synthesis Kit (TaKaRa). BDNF CDS fragments were amplified and inserted into the Fugw vector. The primers sequence is as follows: PF: 5′-GGCGGATCCATGACCATCCTTTTCCTTACTATGG-3′(BamH1 site); 5′-GGCGAATTCCTATCTTCCCCTTTTAATGGTCAGT-3′(EcoR1 site). The vector was packaged to be lentivirus and transfected into the cells by using Lipofectamine 2000 (Thermo) and the instructions for the reagent.

### Sevoflurane Treatment of Mice and Cells

C57BL/J6 mice at postnatal day 6 (P6) (Shanghai SLAC Laboratory Animal, Zhangjiang, Shanghai, P. R. China) were used for sevoflurane treatment. The protocol was approved by the Standing Committee on Animals at Shanghai Ninth People's Hospital, Shanghai, China. The mice received the sevoflurane anesthesia as described in our previous studies (33, 34). The mice were allowed to totally recover from the anesthesia. Each of the mice was euthanized via decapitation at the end of the sevoflurane administration on P8 and the hippocampus tissue were then harvested. Specifically, the cells were treated with 4.1% sevoflurane for 2 h daily at day 4, 5, and 6 after the start of neural differentiation to mimic the clinical several times anesthesia. The cells were harvested at day 7 during the neural differentiation, at which there're many NPCs (26). In some experiments, the cells were transfected with BDNF 12 h before the sevoflurane treatment.

### Flow Cytometry Studies

The cells were suspended in PBS for flow cytometry analysis by using FACS Calibur (BD Biosciences, USA) operating at 488 nm excitation with standard emission filters. Fluorescence noise baseline was referenced with the 46C mESCs. Flowjo software was used to analyze the results.

### Quantitative Real-Time PCR (qRT-PCR)

Total RNA was isolated using RNAiso Plus (TaKaRa). For miRNA, cDNA inverse transcription was carried out with the TIANScript RT Kit (Tiangen, China). qRT-PCR primers of miRNA were purchased (RiboBio, China). mRNA was inverse transcribed to cDNA using cDNA Synthesis Kit (TaKaRa). The primers for detecting mRNA or LncRNA level are as follows:

Rik-203:PF:5′-CATCACTTGGACCATGGACACTAAT-3′, RF:5′-GAATCCTATACACATGAATGCAGAA-3′; Nestin:PF:5′-GAATGTAGAGGCAGAGAAAACT-3′, RF:5′-TCTTCAAATCTTAGTGGCTCC-3′; Sox1:PF:5′-GTTTTTTGTAGTTGTTACCGC-3′, RF:5′-GCATTTACAAGAAATAATAC-3′;

GAPDH:PF: 5′-ATGACATCAAGAAGGTGGTG-3′,

RF: 5′-CATACCAGGAAATGAGCTTG-3′.

### Nucleus and Cytoplasm Extraction

In order to detect the distribution of LncRNA Rik-203 in the NSCs, we performed the nucleus and cytoplasm extraction studies according to the previous study (35). Purification and analysis of cytoplasmic and nucleus RNA was performed by using qRT-PCR.

### Luciferase Reporter Assays

pGl3-cm vector was used to construct the 3′UTR luciferase reporter. BDNF 3′UTR fragment was amplified from mESCs DNA.

The PCR primers are as follows:

For miR-466l-3p binding sites UTR region: PF:5′-GGCGTCGACTGAACTGCATGTATAAATGAAGTTT-3′; PR:5′-GGCTCTAGAAATTGGTACACTTAAATAGAACCTG-3′.

Mutant UTR reporter vector was further obtained from the 3′ UTR luciferase reporter by replacing the 6 base pair (bp) miRNA seed sequence by using the QuikChange Site-Directed Mutagenesis Kit (Stratagene, USA).

For analysis, 3T3 cells (2 × 10 ^4^ cells/well of 48-wells plate) were transfected with 200 ng of the 3′UTR luciferase reporter or mutant one, 5 ng Renilla vector, and 20 pmol of miR-466l-3p mimics or miRNA control mimics (Ribobio). Forty-eight hours later cells were harvested to perform the luciferase level using the Dual Luciferase Assay kit (Promega, USA) and SpectraMax M5 microplate reader (Molecular Devices, USA).

### Western Blot

Cells or tissues were lysed by RIPA buffer to obtain the protein for electrophoresis. Protein was transferred onto the PVDF membrane (BioRed, USA). Then incubated the primary antibodies: GAPDH (ab8245, Abcam) uased for normalizing whole protein levels, and BDNF (ab10505, Abcam). Enhanced chemiluminescence (ECL) substrate (Thermo) was used to visualize the protein expression signaling.

### RNA Pull-Down Assay

1 × 10^8^ mESc-derived NSCs were used for the studies. Full-length C130071C03Rik and the antisense RNA were transcribed into the cells using T7 RNA polymerase. Fifty pmol of C130071C03Rik, or C130071C03Rik's antisense RNA, was labeled using desthiobiotin and T4 RNA ligase via a PierceTM RNA 3′End Desthiobiotinylation Kit (Thermo). The RNA pull-down assay was performed according to the PierceTM Magnetic RNA-Protein Pull-Down Kit (Thermo) and parts of the experiments were performed in the core facilities in Yingbiotech (Shanghai, China). In addition, the cells were briefly lysed with Pierce IP Lysis Buffer, and incubated on ice for 5 min. The lysates were centrifuged at 13,000 × g for 10 min, and the supernatant was transferred to a new tube for further analysis. The labeled RNA was added to 50 μL of beads, and incubated for 30 min at room temperature with agitation. The RNA-bound beads were incubated with the lysates for 60 min at 4°C. The RNA-Binding microRNAs were washed and eluted, and the binding microRNAs were detected using qRT-PCR. Primers for the qRT-PCR analysis of miRNA include the following list. For miR-466l-3p: Primer of Stem-loop reverse transcription:

5′-GTCGTATCCAGTGCGTGTCGTGGAGTCGGCAATTGCACTGGATACGACTCTTATG−3′, Primers for qRT-PCR: PF: 5′- ACGCAGATACATACACGCACA−3′, RF: 5′- AGTGCGTGTCGTGGAGTCG -3′.

### Statistics Analysis

The data were presented as mean ± standard deviation (SD) with more than three independent experiments. The significance of statistics was determined by a Student's *t*-test or one-way ANOVA. ^*^ and # *p* < 0.05, ^**^ and ## *p* < 0.01, ^***^ and ### *p* < 0.001. We used the Graph Pad (Software Inc., San Diego, California, USA) to evaluate all of the study data.

## Results

### Rik-203 Decreased by Sevoflurane and Is Critically Involved in the Neural Differentiation

**Figure 1:**
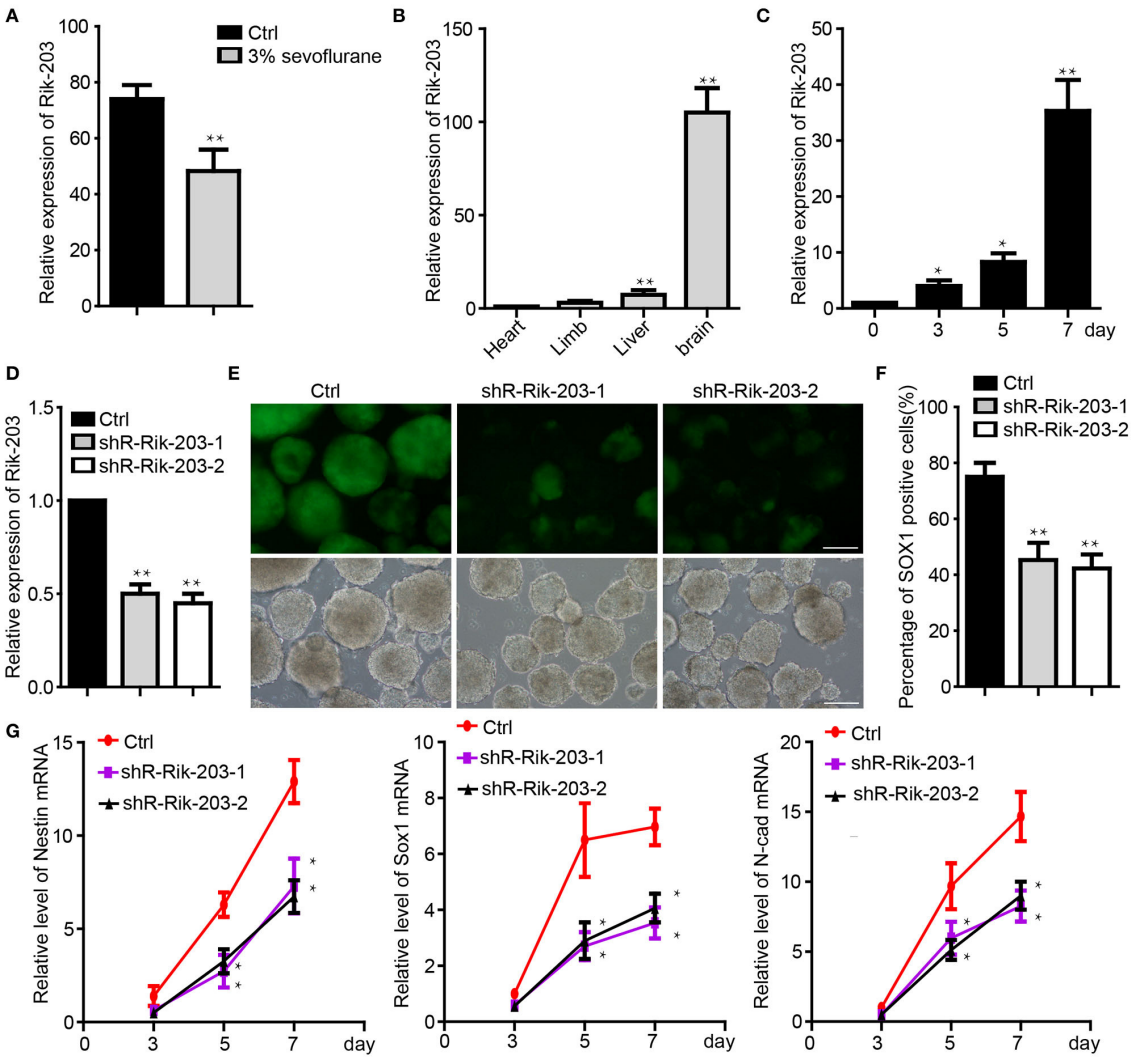
Rik-203 regulatese neural differentiation. **(A)** Sevoflurane decreased the Rik-203 level in the mice hippocampus (*n* = 6). **(B)** qRT-PCR indicated that Rik-203 level is highest in brain compared with other tissues of mice. **(C)** Level of Rik-203 expression during the neural differentiation from ESCs to NSCs. **(D)** Knockdown of the Rik-203 expression by shRNAs and repressed the neural differentiation **(E)**. **(F)** Flow cytometry assay showed the proportion of Sox1-GFP positive cells was repressed by Rik-203 knockdown. **(G)** qRT-PCR showed that levels of Sox1, Nestin, N-cadherin were decreased through Rik-203 knockdown. The scale bar represents 100 μm. Ctrl means control; *p < 0.05, **p < 0.01.

In our previous study, we found that LncRNA Rik-203 contributes to anesthesia induced inhibition of neural differentiation (26). Rik-203 expression level was significantly lower in the hippocampus of mice exposed with 3% sevoflurane 2 h daily for 3 days (Figure 1A). Moreover, LncRNA Rik-203 was especially enriched in the mouse brain than in other tissues, such as limb, heart and liver (Figure 1B). We then performed the induction of neural differentiation from mouse embryonic stem cells (ESCs) 46c and found that the amount of Rik-203 was also upregulated significantly during the neural differentiation from mESCs to neural stem cells (NSCs) (Figure 1C). In the contrary we repressed the process of neural differentiation derived from 46C mESCs, the Sox1-promoter GFP transgenic ESCs (36), by treating the cells with sevoflurane, which is a widely used anesthetic and induces neural development neurotoxicity in developing brain (37–39). To investigate whether Rik-203 could regulate the neural differentiation, we knocked down the Rik-203 (Figure 1D) and found that the neural differentiation form mESCs was inhibited by Rik-203 knockdown (Figure 1E). Flow cytometry assay confirmed our findings in Figure 1E, and the proportion of Sox1-GFP positive cells was indeed repressed by Rik-203 knockdown (Figure 1F). Additionally, the expression of Nestin, Sox1, and N-cadherin, which are critical neural development marker genes, was significantly decreased in Rik-203 knockdown groups (Figure 1G).

### Rik-203 Targeted the miR-466l-3p During the Neural Differentiation

**Figure 2:**
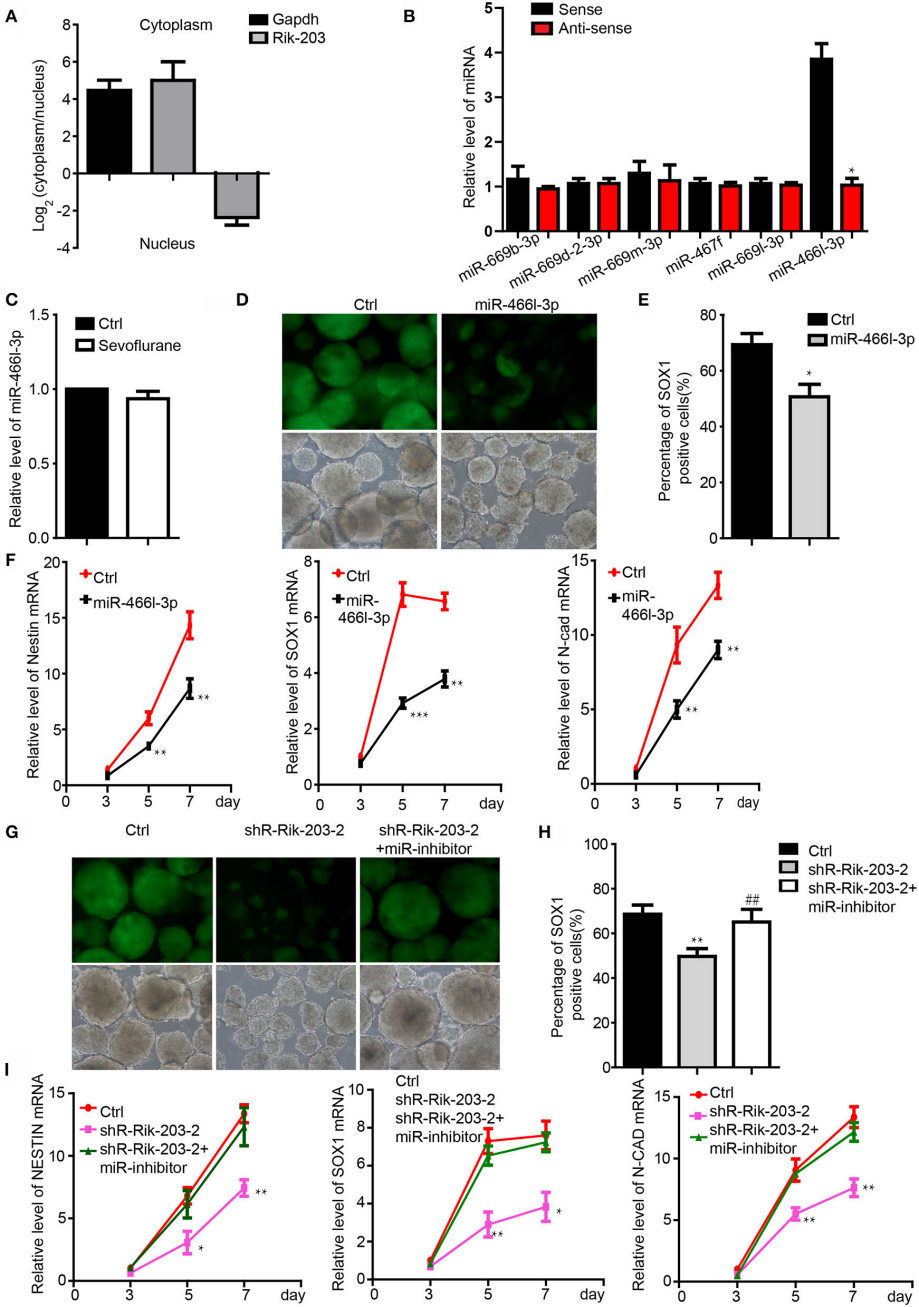
Rik-203 targeted the miR-466l-3p during the neural differentiation. **(A)** Cytoplasmic and nucleus distribution of Rik-203 was detected by RT-PCR and showed that there were higher Rik-203levels in the cytoplasm. **(B)** RNA pull-down assay showed that miR-466l-3p bind to Rik-203. **(C)** Sevoflurane could not affect the level of miR-466l-3p in mice hippocampus. **(D)** Overexpression of miR-466l-3p significantly repressed the neural differentiation. **(E)** Flow cytometry assay also confirmed the inhibition of proportion of Sox1-GFP positive cells. **(F)** qRT-PCR showed the downregulation of NSCs related genes Sox1, Nestin, N-cadherin by miR-466l-3p. **(G)** Rescue experiment showed that inhibition of miR-466l-3p restored the neural differentiation repression caused by Rik-203 knockdwon. miR-inhibitor means the miR-466l-3p inhibitor that is the chemically modified RNA single chain competing with mature miR-446l-3p. Ctrl means the plko-tet-on vector and miR-inhibitor control. **(H)** Flow cytometry assay also indicated the inhibition of mir-466l-3p recued the neural differentiation in **(G)**. **(I)** Detection of the NSCs related genes expression in the rescue experiments. The scale bar represents 100 μm. *p < 0.05, **p < 0.01, ***P < 0.001; ** or ^*##*^*p* < 0.01.

We examined the downstream mediator of Rik-203 during neural differentiation. There are more Rik-203 distributing in the cytoplasm than in the nucleus (Figure 2A), which indicates the competing endogenous RNAs (ceRNA) function of Rik-203 in the cytoplasm. Bioinformatics assay and RNA pull down analysis showed that mmu-miR-466l-3p could bind with the Rik-203 (Figure 2B). Additionally, sevoflaurne treatment did not alter miR-466l-3p expression (Figure 2C), which further suggested the regulatory function of Rik-203 over ceRNA. Overexpression of miR-466l-3p significantly repressed the neural differentiation (Figures 2D,E) as well as the expression of NSCs markers, namely Sox1, Nestin and N-cadherin (Figure 2F). In addition, subsequent rescue experiments found that inhibition of miR-466l-3p by specific miRNA inhibitors could restore the repressed neural differentiation (Figures 2G,H) as well as the decrased NSCs related gene expression (Figure 2I) caused by Rik-203 knockdown. These findings suggest that Rik-203 can bind to miR-466l-3p during the neural differentiation process.

### BDNF Is Directly Targeted by miR-466l-3p and Mediates Neural Differentiation

**Figure 3:**
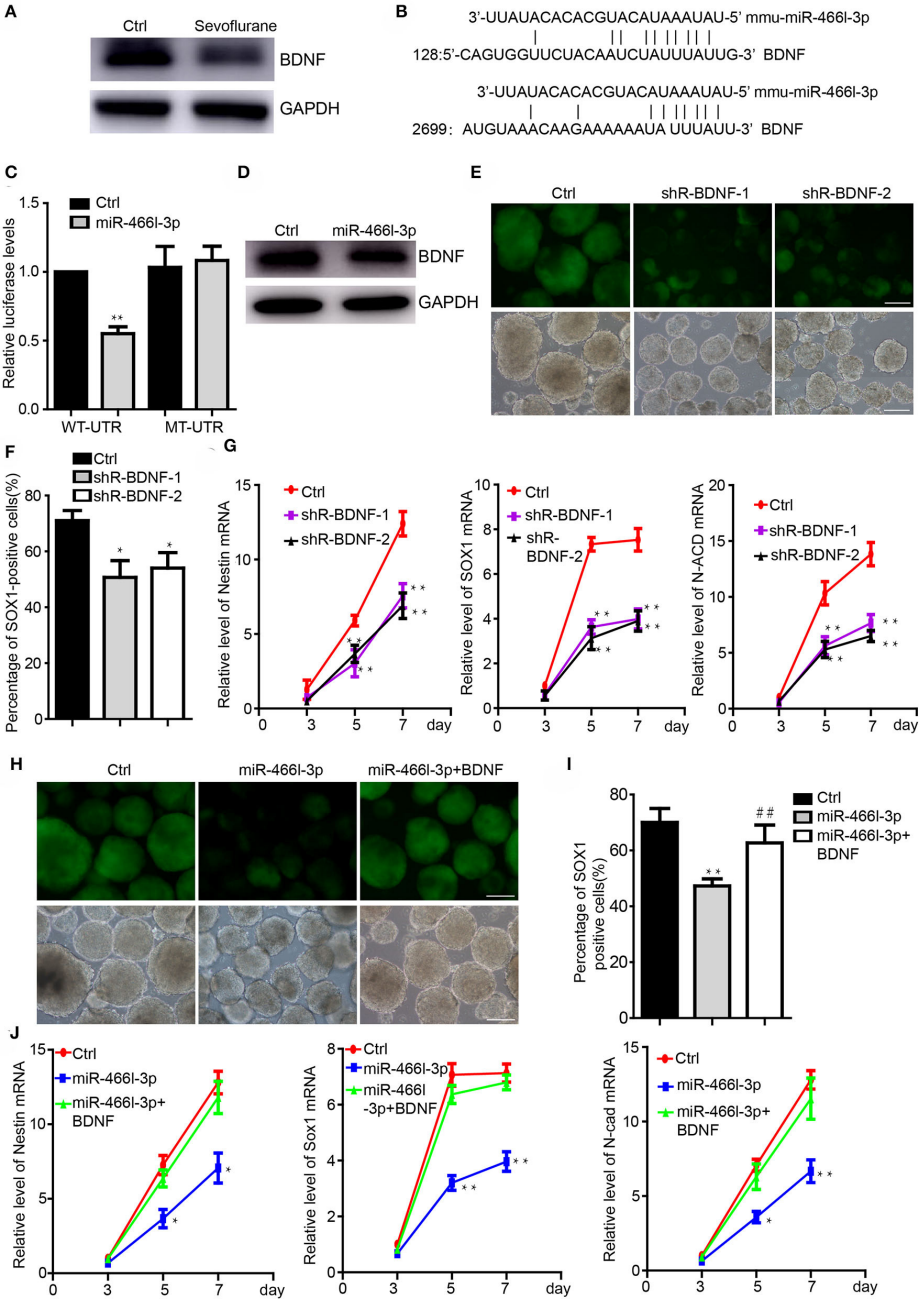
MiR-466l-3p targeted BDNF to mediate neural differentiation. **(A)** Western blot indicated the downregulation of BDNF cause by sevoflurane treatment in mice hippocampus (*n* = 6). **(B)** Target validation of the binding of BDNF 3′UTR by miR-466l-3p. **(C)** Luciferase report assay indicated that miR-466l-3p targeted wild-type BDNF 3′UTR but not mutant UTR. **(D)** Overexpression of miR-466l-3p decreased the protein level of BDNF. **(E)** Knockdown of the BDNF inhibited the neural differentiation. **(F)** Flow cytometry assay confirmed the downregulation of BDNF repressed the proportion of Sox1-GFP positive cells. **(G)** Detection of the NSCs related genes Sox1, Nestin, N-cadherin expression by qRT-PCR. **(H)** Overexpression of BDNF blocked the neural differentiation caused by miR-466l-3p, which was also confirmed in **(I)**. **(J)** Detection of the expression of Sox1, Nestin, and N-cadherin in the **(H)**. The scale bar represents 100 μm. *p < 0.05, **p < 0.01; ** or ^*##*^*p* < 0.01.

We next addressed the interaction of Brain derived neurotrophic factor (BDNF) and miR-466l-3p under anesthesia exposure. Brain derived neurotrophic factor (BDNF) is involved in the differentiation from iPSCs to NSCs (40) while promoting the growth of neurons and NSCs (41). Sevoflurane, on the other hand, has been shown to significantly inhibit the BDNF expression and cognitive functions (39). We detected the downregulation of the BDNF in cells treated by sevoflurane during the neural differentiation (Figure 3A). MiR-466l-3p is predicted to target BDNF(Figure 3B) by TargetScan (42), miRBase (43). To investigate whether BDNF could be directly targeted by miR-466l-3p, we engineered luciferase reporters with either the wild-type 3′ UTRs or mutant UTRs with deletion of 6 base pair (bp) miRNA seed sequence binding sites. A scrambled control with no homology to the mouse genome was used to control the nonspecific effects of endogenous miRNA expression. Overexpression of miR-466l-3p repressed the luciferase expression. In contrast, mutant reporter was not repressed by miR-466l-3p (Figure 3C). Overexpression in the NSCs also induced the downregulation of BDNF (Figure 3D). These data indicated that 3′UTR of BDNF was directly targeted by miR-466l-3p. Knockdown of the BDNF could inhibit the neural differentiation (Figures 3E,F) while repressing the expression of Sox1, Nestin and N-cadherin (Figure 3G), which was similar with the phenomenon of the overexpression of miR-466l-3p. Additionly, we performed the rescue experiments to study whether miR-466l-3p/BDNF pathway could function as the regulator of neural differentiation. Indeed, overexpression of BDNF significantly mitigated the repression of neural differentiation caused by miR-466l-3p overexpression (Figures 3H,I). The expression of NSCs markers Sox1, Nestin and N-cadherin expression was also restored by BDNF in the miR-466l-3p overexpressing cells (Figure 3J). These findings together suggest that BNDF is the direct target of miR-466l-3p during sevoflurane exposure and mediates neural differentiation.

### BDNF Restored the Neural Differentiation Repression Caused by Rik-203 Downregulation

**Figure 4:**
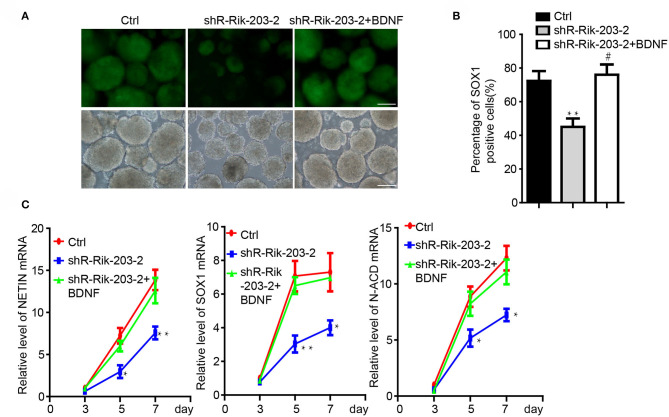
BDNF restored the repression of neural differentiation caused by Rik-203 downregulation. **(A)** Overexpression of BDNF restored the neural differentiation repressed by Rik-203 knockdown. **(B)** Flow cytometry assay showed that BDNF rescue the proportion of Sox1-GFP positive cells repressed by Rik-203 knockdown. **(C)** The expression level of Sox1, Nestin and N-cadherin is also restored by BDNF in Rik-203 knockdown cells. The scale bar represents 100 μm. *p <0.05, **p <0.01; or ^#^*p* <0.05.

Knockdown of BDNF induced the neural differentiation repression, which was similar with the phenomenon following Rik-203 knockdown. miR-466l-3p has also been confirmed to be the downstream target of Rik-203. Next we found that overexpression of BDNF could be the downstream target of RIk-203 to rescue the repression of neural differentiation cause by Rik-203 (Figures 4A,B) and also the expression of NSCs marker genes expression (Figure 4C).

## Discussion

In the current studies, we were able to show that sevoflurane decreased levels of LncRNA Rik-203 in the brain tissues of the mice. Such reductions resulted in the inhibition of neural differentiation via the miR-466l-3p /BDNF signaling. These data also suggest that Rik-203 would involved in the underling mechanism for sevoflurane anesthesia neurotoxicity.

LncRNAs are known to function as epigenetic modulators (44) andplay crucial roles in developmental and neurodegenerative diseases (45). Among five 5 transcripts (splice variants) in LncRNA Riken,LncRNA Rik-201 and Rik-203 have functional role in regulating neural differentiation. Previous studies have shown that anesthetics may regulate LncRNAs expression (32). Sevoflurane increased the level of LncRNA Gadd45a in the rat hippocampus neural stem cells and induced neurotoxicity (46). Our findings showed that Rik-203 was upregulated gradually during the neural differentiation and downregulated by sevofalurne treatment in mice brain. Rik-203 is mainly located in the cytoplasm to function as the ceRNA and to inhibit downstream miR-466l-3p effects of repressing the neural differentiation. Sevoflurane decreased the levels of Rik-203, which led to the release of the miR-466l-3p from Rik-203. The released miR-466l-3p then decreased the levels of BDNF.These results indicated that Rik-203/ miR-466l-3p/BDNF pathway might contributed to sevoflurane induced neural differentiation process inhibition.

Sevoflurane was also reported to decrease the expression of BDNF and induced cognitive impairment in 18 month-old rats (39). BDNF is critically involved in the differentiation of NSCs from iPSCs (40) and promotes neurons and NSCs growth (41). We found that miR-466l-3p directly targets BDNF, leading to the inhibition of the neural differentiation. BDNF overexpression could restore the neural differentiation repressed by the Rik-203 knockdown or miR-466l-3p overexpression. These data indicated that BDNF construct the Rik-203/miR-466l-3p/BDNF signaling pathway regulating the neural differentiation and mediating sevoflurane neurotoxicity.

There are several limitations in the studies. First, we did not perform *in vivo* relevance studies to investigate the neural differentiation inhibition in miR-466l-3p knockout in mice. However, the *in vitro* findings demonstrated that sevoflurane could inhibit neural differentiation via the Rik-203/miR-466l-3p/BDNF signaling pathway. Second, we also did not perform dose-response curve of sevoflurane on cells and mice in this study. In our previous study, we found that sevoflurane also decreased Rik-203 mRNA levels in the hippocampus tissues of mice in a dose-dependent manner (26).

## Conclusion

We identified the functional role of sevoflurane regulating LncRNA Rik-203 in facilitating neural differentiation and elucidated the underlying miR-466l-3p/BDNF associated molecular pathway mechanisms. Moreover, these findings have identified Rik-203 as a potential target for prevention and treatment of anesthesia neurotoxicity in young mice and children.

## Data Availability Statement

The datasets used and/or analyzed during the current study are available from the corresponding author on reasonable request.

## Ethics Statement

The animal protocol was approved by the Standing Committee on Animals at Shanghai Ninth People's Hospital, Shanghai, P.R. China.

## Author Contributions

LZ and HJ: study concept and design. LZ, ZX, and JY: acquisition of data, analysis, and interpretation of data. LZ and ZX: drafted of the manuscript. LZ, JY, and HJ: obtained funding, administrative, technical, and material support. All authors contributed to the article and approved the submitted version.

## Conflict of Interest

The authors declare that the research was conducted in the absence of any commercial or financial relationships that could be construed as a potential conflict of interest.

## Abbreviations

ESCs: Embryonic stem cells
LncRNAs: Long non-coding RNAs
NPCs: Neural progenitor cells
BDNF: Brain-derived neurotrophic factor.
